# The MDPV Derivative α-PHP Regulates Cellular Differentiation and Triggers Apoptotic Cell Death and Ultrastructural Changes in Murine 3D Neurospheres

**DOI:** 10.3390/molecules31142453

**Published:** 2026-07-13

**Authors:** Fabrizio De Luca, Cinzia Brenna, Marta Bassi, Sabrine Bilel, Adolfo Gregori, Carlo Alessandro Locatelli, Luca Maria Neri, Raffaella Adami, Daniele Bottai, Matteo Marti, Elisa Roda

**Affiliations:** 1 Department of Biology and Biotechnology “L. Spallanzani”, University of Pavia, 27100 Pavia, Italy; 2 Department Chemical, Pharmaceutical and Agricultural Sciences, University of Ferrara, 44121 Ferrara, Italy; brncnz@unife.it; 3 Laboratory for Technologies of Advanced Therapies “LTTA”—Electron Microscopy Center, University of Ferrara, 44121 Ferrara, Italy; luca.neri@unife.it; 4 Department of Translational Medicine, Section of Legal Medicine and LTTA Centre, University of Ferrara, 44121 Ferrara, Italy; marta.bassi@unife.it (M.B.); sabrine.bilel@unife.it (S.B.); matteo.marti@unife.it (M.M.); 5 Department of Scientific Investigation (RIS), Carabinieri, 00191 Rome, Italy; adolfo.gregori@carabinieri.it; 6 Laboratory of Clinical & Experimental Toxicology, Pavia Poison Centre, National Toxicology Information Centre, Toxicology Unit, Istituti Clinici Scientifici Maugeri IRCCS, 27100 Pavia, Italy; carlo.locatelli@icsmaugeri.it; 7 Department of Translational Medicine, University of Ferrara, 44121 Ferrara, Italy; 8 Department of Pharmaceutical Sciences, Section of Pharmacology and Biosciences, University of Milan, Via Balzaretti 9, 20133 Milan, Italy; raffaella.adami@unimi.it (R.A.); daniele.bottai@unimi.it (D.B.); 9 Collaborative Centre for the Italian National Early Warning System, Department of Anti-Drug Policies, Presidency of the Council of Ministers, 00184 Rome, Italy

**Keywords:** synthetic cathinones, α-PHP, new psychoactive substances (NPS), 3D neurospheres, neural stem/progenitor cells (NSPC), MAP, GFAP, Caspase-3, TEM

## Abstract

Cumulative reports of psychiatric and neurological outcomes due to synthetic cathinones continue to raise public concern. However, the understanding of the neurotoxic mechanism of action is still poorly understood, particularly for the under-explored αPHP, one of the main MDPV derivatives. In particular, the effects of this synthetic drug on neural stem/progenitor cell cultures are still unknown. Therefore, in the proposed in vitro study, the effects of increasing αPHP concentrations (50–2000 μM) on cell morphology, neuronal/glial differentiation, cell death pathways, and ultrastructure have been evaluated after exposure in murine 2D NSPCs and 3D neurospheres using complementary techniques, i.e., phase contrast microscopy, immunocytochemistry, confocal microscopy, and transmission electron microscopy. We observed that αPHP was able to induce a dose-dependent neurotoxic and neuromodulatory effect in murine 2D NSPC cultures and a 3D neurosphere model, affecting neuronal/glial differentiation, activating the apoptotic pathway, and inducing morphological and ultrastructural changes. The present study could pave the way for a broadened knowledge of synthetic cathinone (SCs) toxicology, needed to establish the right treatment for novel psychoactive substance (NPS) exposure and the possible consequences for public health.

## 1. Introduction

In recent years, the world has encountered an emerging problem, raising more and more striking proportions: the spread of New Psychoactive Substances (NPS), characterized by specific pharmacological and toxicological characteristics, strongly threatening consumers’ health, hence representing an emerging public health issue and a major concern for National Regulatory Agencies and International Health Regulatory Bodies. In fact, in recent decades, an exponential growth in the appearance of NPS in the illicit drug market occurred, reaching 1124 substances stated to the United Nations Office on Drugs and Crime by December 2021 [1], with a consequent escalation of case reports of acute, sometimes lethal, intoxications and adverse effects [2,3,4,5]. Regrettably, the currently available data on potency, pharmacological effects, and risk profiles of NPSs are neither exhaustive nor conclusive; nonetheless, the toxicity of NPSs is extremely worrying, since they are capable of causing deadly toxidromes or even severe medical risks, including irreversible functional alterations of the Central Nervous System (CNS) [6], also damaging peripheral organs. The difficult clinical management of NPS toxidromes [4,5,7,8,9,10,11] is also hindered by the absolute lack of knowledge concerning the medium and long-term consequences, including their abuse potential, dependence/withdrawal outcomes, and also the likely neurotoxic action accountable for triggering permanent brain injuries/deficits. Among recently monitored NPSs, 162 molecules belong to the class of synthetic cathinones (SCs), β-keto-amphetamine analogues, which are one of the most widespread designer drugs proposed by the global illicit market [12,13,14,15,16]. The main mechanism underlying the SCs’ pharmacological actions is the dysregulation of the central monoaminergic pathways, frequently correlated with fatal and non-fatal intoxications [17,18,19,20]. SCs, usually branded “not for human consumption” or “jewellery cleaner” and “phone screen cleaner”, commonly known as “legal highs”, “bath salts” and “plant food” with the aim at persuading with a notion of safe and legal substances, display psychostimulant properties similar to those triggered by conventional drugs, i.e., cocaine, MDMA and amphetamine [18,21]. α-PHP, a less common analogue of αPVP, is one of the pyrovalerone derivatives (-pyrrolidinophenones) chemically characterised by the presence of a long side chain linked to the α-carbon and a pyrrolidine ring [22,23]. This synthetic stimulant drug was originally developed in the 1960s, when the United States Patent Office published a document concerning the chemical synthesis methods for α-pyrrolidine ketones and related novel substances, including α-PHP. Since then, it has been commonly linked to polydrug abuse cases, triggering typical SCs-induced stimulant effects such as sociability, euphoria, intensified sensory experiences, reduced appetite, and increased energy [21,24]. Particularly, severe adverse effects have been recorded, including but not limited to restlessness, agitation, psychosis, hallucinations, bruxism, tachycardia, and seizures [17,20,24,25,26,27,28]. In recent years, numerous in vivo investigations have been conducted to characterize the neurotoxicity mechanisms of action underlying the SCs-elicited adverse clinical effects, including disruption of monoaminergic neurotransmitter pathways, inflammation, respiratory alterations, cardiovascular damage, alterations in thermoregulation, and oxidative stress [5,21,29,30]. The SCs’ pharmacological activity has also been explored, highlighting the α-PHP-elicited selective and potent inhibition of dopamine and norepinephrine reuptake, instead revealing negligible consequences on serotonin transporter. Moreover, the α-PHP incapability to boost neurotransmitter release was also described [4,21,26,31]. Recently, in vitro research focused on SCs-induced neurotoxicity, highlighting oxidative stress, neuronal Ca^2+^ homeostasis disruption, and mitochondrial dysfunction as key outcomes [4,5,32]. However, despite already existing data, an urgent need emerged for an exhaustive identification/comprehension of the SCs’ complex neurotoxic mechanisms, particularly for under-investigated compounds, for example, α-PHP [9,33,34]. Recently, we studied the effect of α-PHP on undifferentiated neural stem/progenitor cell cultures (NSPCs), thus simulating the situation of very early developmental phases, when the stem cells located in the developing central nervous system are rapidly dividing [35], and we demonstrated in vitro the detrimental effects of this drug, revealing a dose-dependent significant decrease in cell viability, proliferation, and clonal capability, together with resting membrane potential depolarisation, and apoptotic/necroptotic/autophagic pathway activation [35].

Hence, based on the pivotal notion that (i) neurogenesis generates newborn neurons from NSPCs, located in specific and restricted CNS areas, occurring beyond development during the adult life in mammals [36,37] and (ii) the CSN is particularly vulnerable to novel psychoactive substances, and alterations of adult neurogenesis elicits several long-lasting and potentially irreversible impairments, involving neuroplasticity change, in the current paper, we explore α-PHP neurotoxic effects using a variant of the previously employed in vitro model, i.e., neural stem/progenitor cell cultures (NSPCs), obtained from C57BL/6 mice subventricular zone, one of the neurogenic niches that are present in the adult murine brain, but focusing on the differentiation capacity of these cells [35,37,38]. NSPCs, characterized by peculiar staminal properties, are in fact cells capable of self-renewal and differentiation into multiple neural lineages during embryo development and the perinatal period, essential for brain development and physiological functions, expressing the ability to differentiate into the three major neural cell lineages, i.e., astrocytes, oligodendrocytes, and neurons [39]. 

Hence, in the present work, we assess α-PHP neurotoxic effects on differentiated NSPCs in 2 and 3D models. In particular, we evaluated potential α-PHP-induced injury in neural cell monolayers and neurospheres containing NSPCs, apoptotic cells, neurons, oligodendrocytes, and astrocytes [39].

In particular, we explored the effects of increasing α-PHP concentrations (50 to 200 µM) on glial and neuronal differentiation, cell death pathway activation, and morphological/ultrastructural alterations, by means of immunofluorescence, confocal microscopy, and transmission electron microscopy (TEM).

## 2. Results

To assess the impact of α-PHP on lineage specification and cell fate regulation, we used neural stem/progenitor cells (NSPCs) derived from the subventricular zone (SVZ), a major neurogenic niche of the adult mammalian brain. Specifically, we investigated how α-PHP modulates the differentiation of neuronal and glial populations, its ability to activate apoptotic pathways within each lineage, and the presence of associated ultrastructural alterations. To obtain a comprehensive evaluation, both monolayer cultures and 3D neurosphere systems were used, enabling the analysis of α-PHP effects across distinct cellular environments and organisational levels.

Based on previous findings [35], α-PHP concentrations ranging from 50 to 200 µM were selected for the present analyses. In particular, 100 µM α-PHP was included as representing the sub-toxic concentration identified and effectively used in earlier investigations.

### 2.1. α-PHP Affects NSPCs Monolayer Differentiation

**Figure 1:**
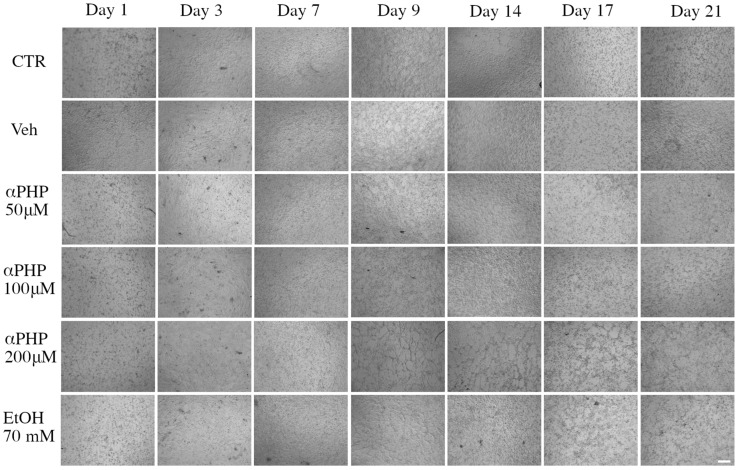
White field images of the cells during the differentiation process. Pictures were taken on days 1, 3, 7, 9, 14, 17, and 21 during different treatments. Scale bar 150 μm. We performed the 2D differentiation of the NSPCs in the presence of different concentrations of α-PHP (50, 100, and 200 μM). By confocal analysis, we detected a reduction in the overall number of MAP2-positive neurons, mainly at 100 and 200 μM after 21 days of differentiation, when compared to Ctrl or vehicle (Figure 2). We also detected a significant change at 14 days of differentiation, but only in the 200 μM experimental group (Figure 3 Panels A–C, and Tables S1–S3). *n* = 3–5.

Phase-contrast morphological analyses showed that no evident alterations were detected at the lowest α-PHP concentrations tested (50 µM and 100 µM). However, by day 9, exposure to the highest dose (200 µM) produced marked changes in the morphology and organisation of NSPCs cultured as monolayers, characterised by regions devoid of cells and displaying a net-like arrangement. A comparable cellular organisation was also observed following ethanol treatment (Figure 1).

Two-dimensional (2D) differentiation represents a more standardized in vitro approach, enabling homogeneous interactions between cells and the substrate, as well as between cells and soluble molecules. In this system, adhesion factors and serum are typically included in the culture medium. In contrast, three-dimensional (3D) differentiation more closely resembles the in vivo situation, as it allows spatial cell–cell interactions within a multicellular structure. In 3D cultures, such as neurospheres, cell–cell contacts and growth factors present in the medium generate concentration gradients that drive and regulate the differentiation process. For these reasons, we decided to further investigate the effects of a-PHP treatment using the 3D NSPCs model.

### 2.2. Synthetic Cathinone α-PHP Differently Modulates the Apoptotic Pathway in Neuronal and Glial Cells of 3D NSPCs Neurospheres

Subsequent analyses aimed at evaluating MAP2 and GFAP expression levels in α-PHP-treated 3D neurospheres focused particular attention on the activation of the apoptotic pathway by assessing Caspase-3 expression and its potential colocalization with the aforementioned neuronal/glial markers.

**Figure 4:**
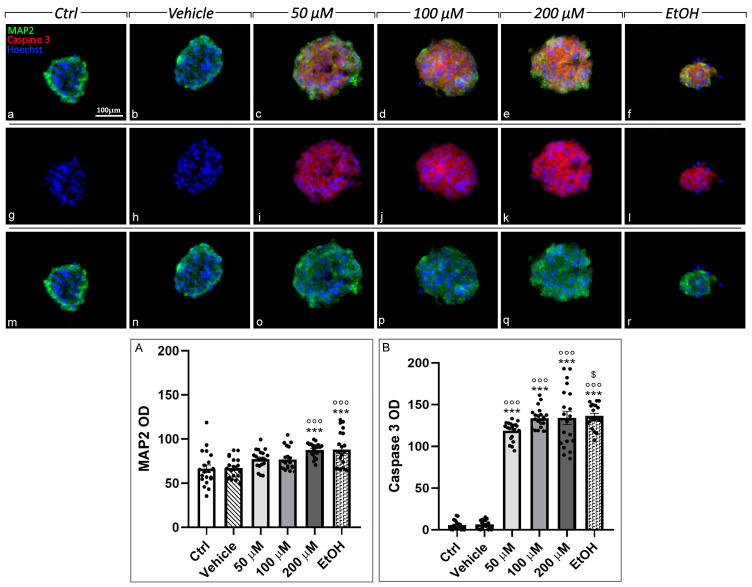
Double immunocytochemical detection of MAP2 (green signal) and Caspase 3 (red signal) by fluorescence microscopy in control (**a**,**g**,**m**), vehicle (**b**,**h**,**n**), EtOH (**f**,**l**,**r**) and differently α-PHP-exposed 3D NSPCs neurospheres, i.e., 50 µM (**c**,**i**,**o**), 100 µM (**d**,**j**,**p**), and 200 µM (**e**,**k**,**q**). DNA counterstaining with Hoechst 33258 (blue fluorescence). Histograms showing the quantitative analyses of MAP2 (**A**) and Caspase 3 (**B**) mean fluorescence intensity per cell. Statistically significant data: *** compared to ctrl; °°° compared to vehicle; $ compared to 50 µM α-PHP. Magnification: 40× (**a**–**r**). Scale bar 100 µm. (***) and (°°°) *p* < 0.001. *n* = 3.

First, regarding MAP2 and Caspase-3 expression, double immunofluorescence staining (Figure 4) showed that cytoplasmatic MAP2 immunopositivity was mainly localised in the outer regions of 3D neurospheres across all experimental conditions, i.e., Ctrl, Vehicle, 50 µM to 200 µM α-PHP, and EtOH. In contrast, cytosolic Caspase-3 immunolabeling was predominantly detected in the core of the spheroids in all treated groups (50 µM, 100 µM, and 200 µM α-PHP, and EtOH). Due to the distinct spatial distribution of these two markers, only a minimal overlap of MAP2 and Caspase-3 fluorescence signals was observed following α-PHP treatment, with the two markers remaining clearly distinguishable under all experimental conditions.

Optical density (OD) analysis of MAP2 expression levels revealed a slight, non-significant increase in immunopositivity, evidenced in 50 and 100 µM α-PHP-treated neurospheres compared to control and vehicle groups. Nonetheless, MAP2 levels significantly increased after exposure to 200 µM α-PHP or EtOH (Figure 4A and Table S4).

Regarding Caspase-3, a highly significant increase in immunoreactivity was determined after α-PHP treatment at all tested concentrations (50 µM, 100 µM, and 200 µM) as well as in the neurosphere exposed to EtOH, compared to control and vehicle groups. Only a small, non-significant additional increase was detected at higher doses (100 µM and 200 µM) relative to 50 µM. Any difference was assessed by comparing the Caspase-3 immunopositivity at the higher concentrations, i.e., 100 µM and 200 µM α-PHP, and in EtOH-exposed neurosphere (Figure 4B and Table S5).

**Figure 5:**
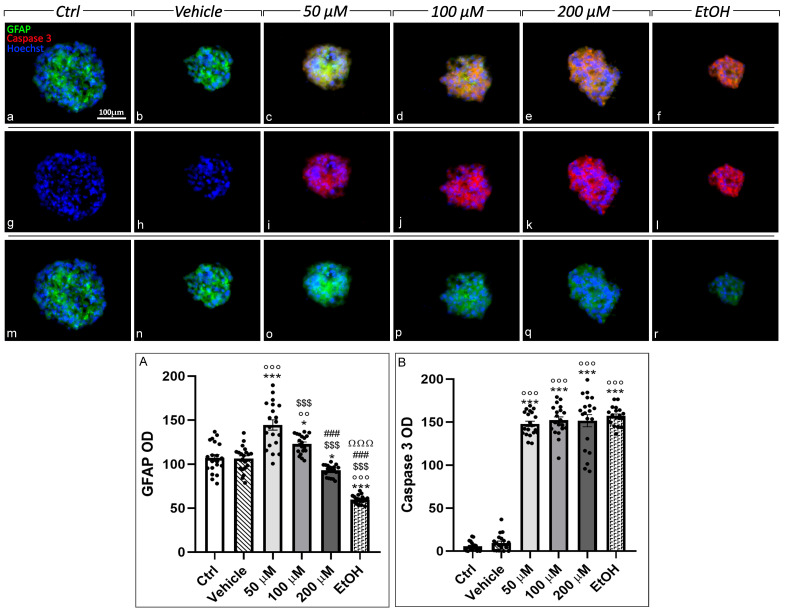
Double immunocytochemical detection of GFAP (green signal) and Caspase 3 (red signal) by fluorescence microscopy in control (**a**,**g**,**m**), vehicle (**b**,**h**,**n**), EtOH (**f**,**l**,**r**), and differently α-PHP-exposed 3D NSPCs neurospheres, i.e., 50 µM (**c**,**i**,**o**), 100 µM (**d**,**j**,**p**), and 200 µM (**e**,**k**,**q**). DNA counterstaining with Hoechst 33258 (blue fluorescence). Histograms showing the quantitative analyses of GFAP (**A**) and Caspase 3 (**B**) mean fluorescence intensity per cell. Statistically significant data: * compared to ctrl; °° and °°° compared to vehicle; $$$ compared to 50 µM α-PHP; ### compared to 100 µM α-PHP; ^ΩΩΩ^ compared to EtOH. Magnification: 40× (**a**–**r**). Scale bar 100 µm. (*) *p* < 0.05; (°°) *p* < 0.01; (***), (°°°), (###), ($$$) and (^ΩΩΩ^) *p* < 0.001. *n* = 3.

Further analyses using double immunofluorescence for GFAP and Caspase-3 (Figure 5) showed that cytosolic GFAP immunopositivity was mainly localised in the neurosphere core across all experimental conditions (Ctrl, Vehicle, 50 µM, 100 µM, 200 µM, and EtOH), exhibiting substantial overlap with cytoplasmatic Caspase-3 immunopositivity in α-PHP-treated and EtOH groups.

OD analysis of GFAP expression revealed that, despite a highly significant increase in immunoreactivity following treatment with 50 µM α-PHP compared with Ctrl and Vehicle, this increase diminished at 100 µM and even further decreased at 200 µM α-PHP and in EtOH group, ultimately falling below control and vehicle levels (Figure 5A and Table S6).

Consistent with the MAP2/Caspase-3 findings, double immunostaining for GFAP and Caspase-3 confirmed a highly significant increase in Caspase-3 immunopositivity following treatment with all α-PHP concentrations (50 µM, 100 µM, and 200 µM) and in the EtOH group compared with Ctrl and Vehicle, with no significant differences among the treated groups (50 µM, 100 µM, 200 µM, and EtOH) (Figure 5B and Table S7). 

### 2.3. Apoptosis, Enhanced Vacuolization, and Mitochondrial Alterations Observed in α-PHP–Exposed Neurospheres by TEM

**Figure 6:**
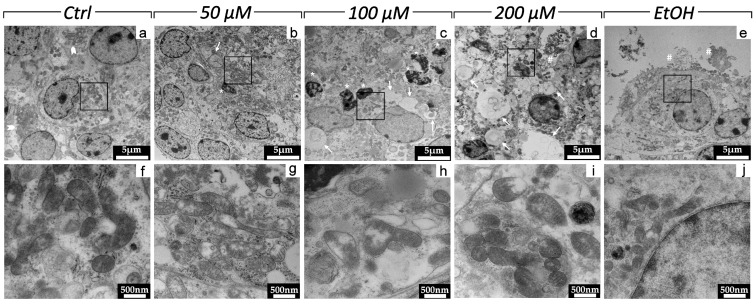
TEM images of control, EtOH, and 50, 100, and 200 µM α-PHP-treated neurospheres, acquired at 2600× (**top row**, **a**–**e**) and 17,500× (**lower row**, **f**–**j**). Drug treatment induces concentration-dependent and progressive disruption of cell structure. Mitochondrial morphology exhibits parallel alterations in response to increasing drug doses. Black squares in the top row indicate the corresponding zoomed-in area of the right one. White arrowheads: Endoplasmic Reticulum (**a**); White asterisks: apoptotic nuclei (**b**–**d**); White arrows: Vacuoles (**b**–**d**); White hashes: extrusion of cytoplasmic structures (**d**,**e**). Scale bars 5 µm (2600×) or 500 nm (17,500×).

Morphological and ultrastructural changes in control and α-PHP-exposed neurospheres were analyzed by TEM (Figure 6).

Control cells exhibited typical ultrastructural morphological features with a regular endoplasmic reticulum, orderly mitochondria, a nucleus with both hetero- and euchromatin, and distinct nucleoli (a;f). We also observed that EtOH (e;j) seems to affect only the cell osmolarity, leading to cell swelling under certain conditions and a leakage of cellular material. 70 mM EtOH-treated cells showed disarrangement of cytoplasm organization, whereas mitochondria and nuclear structures did not reveal significant alterations (e–j). Moreover, in e, the extrusion of cytoplasmic structures outside the cells is also shown. Treatment with 50 μM α-PHP induced the first signs of apoptosis (b), a reduction in mitochondria dimensions and in their cristae density, visible as electron-lucent spaces (g), and a decrease in overall organelle size. The higher concentration of 100 μM α-PHP led to a dose-dependent increase in the number of apoptotic cells (c). This concentration also resulted in a significant enlargement of the intramitochondrial electron-lucent regions (h) and the comparison of multiple cytoplasmic vacuoles (c). After 200 μM α-PHP treatment, vacuoles became even more evident (d), whereas mitochondria did not show increased alterations when compared with the 100µM treatment (i). Apoptotic cells were still present (d). As in EtOH-treated samples, disruption of the cytoplasmic membrane was evident, leading to the leakage of cytoplasmic structures (e).

Micrographs are representative of *n* = 5 fields of view examined per grid across *n* = 2 independent TEM grids for each condition.

## 3. Discussion

The global escalation of NPSs has created a critical public health crisis, with more than 1 thousand distinct compounds identified by late 2021 [1]. This rapid market increase is characterized by a significant knowledge gap regarding the possible pharmacological properties and long-term safety of these drugs. This lack of information complicates patient management, particularly as many novel psychoactive substances are usually associated with systemic organ failure, fatal toxidromes, and permanent CNS damage [17,18,19,20,21]. An important subset of these events is the emergence of synthetic cathinones (SCs), which currently comprises more than 150 identified molecules. As beta-keto-amphetamine analogues, SCs dysregulated central monoaminergic pathways, leading to psychostimulant effects that are comparable to cocaine and MDMA [13,14,15]. Among these, alpha-pyrrolidinohexanophenone (α-PHP) has appeared as a potent pyrovalerone derivative originally synthesized in the 1960s, and its major effects include hallucinations, acute agitation, seizures, psychosis, and tachycardia [17,20,24,25,26,27,28,40]. Recent in vivo investigations characterized the neurotoxicity mechanisms underlying the SC-elicited adverse clinical manifestations, including disruption of monoaminergic neurotransmitter pathways, inflammation, respiratory alterations, alterations in thermoregulation, cardiovascular damage, and oxidative stress [5,21,29,30]. Moreover, from a pharmacological activity point of view, α-PHP-elicited selective and strong inhibition of norepinephrine and dopamine reuptake, whereas it revealed slight effects on the serotonin transporter. Furthermore, the α-PHP’s inability to enhance neurotransmitter release was also previously reported [4,21,26,31]. Parallelly, in vitro data have focused on SCs-induced neurotoxicity, highlighting disruption of neuronal Ca2+ homeostasis, changes in oxidative stress levels, and mitochondrial dysfunction [4,5,32]. In the social context, the use of SCs is linked to a repetitive and intensive administration pattern in which other drugs, including alcohol, are concomitantly used [41]. There is a critical link between neurogenic events and several psychiatric disorders, but the possible effects of novel psychoactive substances on neurogenesis remain unknown. Studies carried out during the last decade indicate that psychoactive substances negatively impact adult neurogenesis, supporting the presence of a mutual relationship between postnatal neurogenesis and addictive behaviours [42,43]. Based on this previous data, we may speculate that SCs could also impact neurogenesis in young subjects. We investigated whether α-PHP affects neural stem cell differentiation, with a primary focus on neuronal differentiation. This was examined using two in vitro models: a conventional 2D cell culture and a more physiologically relevant 3D neurospheres system. This latter condition better mimics organoid-like conditions, as neurospheres are complex structures that include not only neural stem cells but also more differentiated cell types such as neurons, astrocytes, and oligodendrocytes. Together, these two experimental paradigms model later stages of neurogenesis and complement our previous findings [35]. Hence, in the proposed cell study, we investigated the unknown effect of α-PHP on cellular morphology, neuronal/glial differentiation, apoptosis activation, and ultrastructure by using a murine cell-based in vitro model, namely NSPCs. In particular, NSPCs obtained from the SVZ of 8-week-old C57BL/6 mice were used to evaluate the potential toxic effects of α-PHP by assessing selected cellular endpoints: (i) cell morphology and neuronal differentiation in 2D NSPCs, using phase-contrast microscopy and immunofluorescence analysis of MAP2 expression levels by confocal microscopy, respectively; (ii) neuronal and glial differentiation in murine 3D NSPC-derived neurospheres, through immunofluorescence analyses of both MAP2 and GFAP expression levels; (iii) possible colocalization of the apoptotic marker caspase-3 with newly differentiated neuronal and glial cells in the 3D model; and (iv) ultrastructural cellular changes in 3D neurospheres following α-PHP exposure, assessed by transmission electron microscopy. We found that α-PHP induced dose-dependent changes in cell morphology and a parallel reduction in MAP2-immunopositive cells in the 2D NSPCs in vitro model. Similarly, this NPS is able to modulate MAP2 and GFAP expression levels in 3D neurospheres and strongly activate caspase-3 already after the lowest tested dose of α-PHP (i.e., 50 µM), as well as impact the normal cellular ultrastructural organisation of our 3D in vitro model following NPS exposure. Although no alterations in cell morphology were observed following treatment with low and intermediate α-PHP concentrations (i.e., 50 µM and 100 µM), exposure to high doses (200 µM) resulted in marked changes in cell morphology and organisation, with a rearrangement of the 2D in vitro model after 9 days. Parallelly, a significant reduction in the number of MAP2-positive neurons was observed after 14 days of differentiation following exposure to 200 µM α-PHP. However, even comparable, or lower concentrations (i.e., 100 and 200 µM) were able to negatively modulate the expression levels of this neuronal marker following prolonged differentiation (21 days). A substantial body of literature highlights the neurotoxic effects of synthetic cathinones (SCs), indicating that these NPS can cross the blood–brain barrier (BBB), act as psychostimulants, and modulate neurotransmitter levels in different brain regions [9,11,44,45,46]. In addition to the toxic effects observed in in vitro models and in laboratory animals (including mice, rats, and zebrafish larvae), cases of intoxication and fatalities have also been reported in humans. These toxic effects are often time- and dose-dependent, with varying degrees of injury depending on the specific SC tested, suggesting a close relationship between molecular structure and biological activity [5,47,48,49]. This modulatory effect on cellular differentiation observed in the 2D model changes markedly when assessed by immunofluorescence studies in 3D neurospheres. Indeed, low doses of α-PHP lead to a slight increase in MAP2 expression levels in this 3D model, while higher concentrations of the drug (200 µM) result in a significant increase in MAP2 immunopositivity, along with a concomitant decrease in GFAP expression levels. These findings are consistent with the previous literature showing that cell–cell interactions enhance neural stem/progenitor cells’ survival, proliferation, and neuronal differentiation through the overexpression of neurotrophic factors and gap junctions [50]. A similar trend, characterised by increased MAP2 levels and decreased expression of the glial marker GFAP, is also observed in the positive control group treated with ethanol. Notably, exposure to neurotoxic substances has been shown to induce divergent cell lineage trajectories during differentiation, leading to alterations in gene expression profiles that are critical for the determination of cell fate. [51]. Similarly, exposure to this NPS is able to strongly activate the apoptotic pathway, as evidenced by a significant increase in caspase-3 activation already at the lowest tested α-PHP dose (50 µM). Moreover, the expression levels of this apoptotic marker remain relatively unchanged even at higher concentrations (i.e., 100 µM and 200 µM). These findings are consistent with previous results obtained in the 2D model, where an increase in caspase-3 expression levels was observed in NSPCs following exposure to different concentrations of α-PHP, accompanied by alterations in the mitochondrial network, which were already significant at 100 µM. [35]. It has to be mentioned that the mitochondrial integrity loss could lead to the formation of vacuoles and autophagic vesicles. The formation of these subcellular structures is a key indicator of autophagic pathway activation, evidencing the initiation of a mitochondrial-mediated autophagic mechanism [52]. In line with this data, in the present study, we revealed α-PHP-induced dose-dependent ultrastructural changes in 3D NSPC-derived neurospheres cells. TEM analysis revealed clear mitochondrial ultrastructural alterations in treated neurospheres compared to control samples, indicative of mitochondrial damage and impaired organelle homeostasis. Such alterations are commonly associated with mitochondrial stress and are known to trigger alternative cellular responses beyond classical apoptotic pathways [53,54]. In particular, damaged mitochondria can activate quality-control mechanisms such as mitophagy to preserve cellular viability, while persistent mitochondrial dysfunction has also been linked to non-apoptotic forms of cell death, including ferroptosis, characterized by oxidative stress and specific mitochondrial morphological changes [55,56]. Although the present ultrastructural observations do not allow a definitive identification of the underlying pathway, the observed mitochondrial features are consistent with stress responses previously described in TEM-based studies. Notably, despite the evident mitochondrial damage, no increase in apoptotic features was observed when comparing neurospheres treated with 100 µM and 200 µM α-PHP. This data supports the hypothesis that non-apoptotic stress response mechanisms may contribute to the observed morphological pattern [57]. It has already been shown that psychostimulant drugs, including SCs, disrupt mitochondrial dynamics (fusion/fission balance) and impair mitophagy, leading to the accumulation of dysfunctional mitochondria, cytochrome c release, and activation of apoptotic pathways; additionally, they alter intracellular calcium homeostasis and endoplasmic reticulum–mitochondria crosstalk, further exacerbating cellular stress and promoting cell death [58,59,60]. Several in vivo and in vitro studies have investigated the toxic effects of synthetic cathinones, particularly methylone, MDPV, mephedrone, and α-PVP, whereas the toxic effects and mechanisms of action of α-PHP remain poorly explored. In this context, our study contributes to filling this gap by using NSPCs as a valuable model to assess the cellular impact of SCs. However, despite the data obtained so far, there is still a strong need for a more comprehensive understanding of the complex neurotoxic mechanisms of NPS, particularly α-PHP. 

Future studies should focus on elucidating the molecular pathways underlying the activation of specific cell death mechanisms, for instance, by investigating different pathways leading to caspase-3 activation, including the evaluation of intrinsic or extrinsic mechanisms by investigating caspase-8 or caspase-9 expression levels, as well as the involvement of pro- and anti-apoptotic molecules (e.g., BAX and BCL2). Furthermore, additional evidence could be obtained through the application of complementary methodologies (e.g., TUNEL and LDH assays) [61,62] to further characterize the occurrence and extent of cell death mechanisms. Additionally, further research should explore in greater detail the activation of autophagic processes through the analysis of autophagy markers (e.g., LC3B) and autophagolysosome-related proteins, as well as the possible involvement of alternative regulated cell death pathways, such as ferroptosis and necroptosis. 

Moreover, although the present study was designed to evaluate the direct neurotoxic effects of α-PHP, the potential contribution of metabolic processes should also be considered. Neural stem/progenitor cells and differentiated neural populations are not metabolically inert. The brain expresses several xenobiotic-metabolizing systems, including cytochrome P450 enzymes (CYPs), flavin-containing monooxygenases, esterases, epoxide hydrolases, and conjugating enzymes, which can locally transform exogenous compounds within neural tissue [63,64]. Consequently, the NSPC-derived neurospheres employed in the present study may retain a limited capacity for xenobiotic biotransformation, partially reproducing extrahepatic metabolic processes occurring in the central nervous system. However, the metabolic competence of ex vivo cellular models is generally lower than that observed in vivo. Drug-metabolizing enzymes, particularly CYP isoforms, are often expressed at low levels in cultured neural cells and may progressively lose activity during prolonged in vitro maintenance [65]. Therefore, metabolic conversion of synthetic cathinones in the neurosphere is expected to be limited compared with hepatic metabolism, and the biological effects observed in the present study are likely driven predominantly by the parent compound rather than by extensive metabolite formation [66]. Nevertheless, the contribution of locally generated metabolites cannot be completely excluded, especially during long-term exposure. Studies using human hepatocytes identified side-chain hydroxylation, oxidation of the pyrrolidine ring to lactam metabolites, ring opening, ketone reduction, and subsequent phase II conjugation reactions as the major metabolic pathways of α-PHP and related pyrrolidinophenone cathinones [22]. The analysis of α-PHP in human urine samples of 13 users revealed a biotransformation of the cathinone, majorly triggered by oxidation of the pyrrolidine ring [67]. Recent preclinical investigations by Bassi et al. demonstrated that α-PHP is rapidly distributed to peripheral tissues and the brain after systemic administration in mice, confirming its ability to reach central nervous system targets. Moreover, the study identified α-PHP together with several metabolites in urine, including dihydro-α-PHP, hydroxy-α-PHP, oxo-α-PHP, dihydro-hydroxy-α-PHP, and hydroxy-oxo-α-PHP, supporting the occurrence of extensive oxidative biotransformation in vivo and revealing sex-related differences in pharmacokinetic profiles and metabolite elimination. These findings suggest that the neurotoxic effects observed in vivo may result from the combined action of the parent drug and its metabolites reaching the brain after systemic metabolism [34]. Consequently, the present 3D neurosphere model serves as a powerful and essential framework for elucidating the precise cellular mechanisms and acute endogenous responses triggered by α-PHP.

## 4. Materials and Methods

### 4.1. Synthetic Cathinone α-PHP

**Figure 7:**
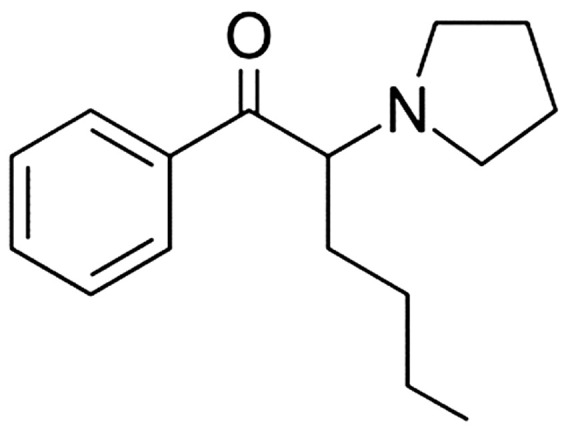
Chemical structure of α-PHP.

The synthetic cathinone α-PHP (Figure 7) was provided by Prof. Matteo Marti (Section of Legal Medicine and LTTA Centre, Department of Translational Medicine, University of Ferrara, 44121 Ferrara, Italy) and acquired from LGC Standards (LGC Standards S.r.l., Sesto San Giovanni, Milan, Italy). α-PHP was then dissolved in phosphate-buffered saline (PBS; Sigma-Aldrich, Milan, Italy) to prepare a 40 mM stock solution and then stored at −20 °C until use.

### 4.2. In Vitro Differentiation Protocols of Murine Neurosphere-Derived NSPCs

NSPCs cells represent a valuable model for studying drug effects on the central nervous system. They are derived from the primary neurogenic niche of the brain, the SVZ. In vivo, adult neural stem cells (aNSCs) located in the lateral subventricular zone (lSVZ) migrate along the rostral migratory stream to the olfactory bulb, where they differentiate mainly into granule and periglomerular interneurons. These neurons primarily belong to the γ-aminobutyric acid (GABA)-ergic lineage, with a small subpopulation being dopaminergic [68,69,70]. In addition, the dorsal region of the SVZ is able to generate a limited number of glutamatergic neurons in the olfactory bulb [70].

When differentiation is performed in vitro, the neuronal/astroglial phenotype depends on the stimuli present in the culture conditions. Our differentiation protocol consists of three main steps: (i) the use of an adhesive substrate, (ii) the withdrawal of growth factors, and (iii) the addition of fetal bovine serum. These conditions promote glial differentiation; however, approximately 15–18% of the cells acquire a neuronal phenotype. Due to the intrinsic regional specification of SVZ-derived cells, these neurons are expected to adopt predominantly an inhibitory fate and to express GABAergic markers [71].

With regard to the 3D differentiation protocol, which is performed in the absence of serum, we expect that the intrinsic specification of SVZ neural stem cells will similarly lead to the predominance of inhibitory neurons. This is consistent with studies performed under serum-free conditions after 14–21 days of treatment [72], in which approximately 45–50% of the differentiated neurons display a GABAergic phenotype. 

In our protocol, neurospheres were mechanically dissociated once they reached an appropriate size (approximately 0.1 mm), and two experimental differentiation assays were performed as described below.

#### Monolayer and 3D Neurosphere Differentiation Assays

For the 2D differentiation protocol, dissociated neurospheres were plated at a density of 40,000 cells per well in a 48-well plate containing a 10 mm Cultrex-coated round glass coverslip (Tema Ricerca, Italy). Cells were maintained for 2 days in proliferation medium (PM) without EGF. The medium was then replaced with PM, lacking both EGF and FGF but supplemented with 1% fetal calf serum. Differentiation was assessed at 7, 14, and 21 days at 37 °C with 5% CO_2_ [73] in the presence of α-PHP at concentrations of 50 μM, 100 μM, and 200 μM [35]. Experimental controls included untreated cultures (ctrl), vehicle controls (vehicle), and a positive control (EtOH 70 mM). Fresh medium was added every 2 days, and ethanol was replenished daily due to its complete evaporation within 24 h. At the end of the treatment, cells were washed with 1×PBS and fixed with 4% paraformaldehyde (PFA) for 10 min at room temperature (RT).

For the 3D neurosphere Differentiation protocol, dissociated neurospheres were plated at a density of 2 × 10^6^ cells in T175 flasks and allowed to grow for 14 days, a time point chosen to ensure an adequate level of differentiation while maintaining a reduced rate of cell death in the presence or absence of α-PHP at 50 μM, 100 μM, or 200 μM. Control treatments included the ctrl, vehicle, and EtOH groups.

### 4.3. Phase-Contrast Microscopy

Micrographs were acquired using the videomicroscope EVOS XL CORE (Life Technologies Italia, Monza, Italy; Life Technologies Europe BV, Bleiswijk, The Netherlands), equipped with a 10× objective.

### 4.4. Microtubule-Associated Protein 2 Staining 2D Experiment

Fixed cells were permeabilised with 0.1% Triton X-100 in PBS for 10 min at RT, followed by incubation with primary antibodies against recombinant microtubule-associated protein, MAP2 (see Table 1), overnight at 4 °C in PBS containing 10% normal goat serum (NGS). Fluorophore-conjugated secondary antibodies were then applied (Alexa Fluor 488-Goat anti-mouse, Immunological Sciences, IS20010). Nuclei were counterstained with 300 nM 4′,6-diamidino-2-phenylindole dihydrochloride (DAPI). All experiments were performed at least three times. For each sample, treatment, and time point, an average of more than 200 cells was counted. Images were acquired using a Zeiss LSM 900 confocal microscope (Oberkochen, Germany) with 10× and 40× oil immersion objectives and 405, 488, and 630 nm lasers. Zen Zeiss software (version 3.0.79.0006) was used for image acquisition, and all parameters were kept constant within each experimental series.

### 4.5. Immunofluorescence and Optical Density Analysis 3D Experiment

Neurospheres were harvested by centrifugation at 500× *g* for 6 min, fixed in 10% formalin for 30 min at RT, and then subjected to sequential washes in PBS and PBS with 0.1% Tween-20. After a final wash, the neurospheres were incubated overnight in 30% sucrose, embedded in OCT compound on dry ice, and sectioned at 8 µm thickness using a cryostat (Leica CM1850). Sections were collected on charged slides and briefly equilibrated in PBS before immunolabeling with selected monoclonal or polyclonal primary antibodies diluted in PBS (Table 1), as previously reported [74]. Briefly, after a 1-h incubation at RT in a dark, humid chamber, sections were washed three times with PBS and incubated for 1 h with the appropriate secondary antibodies diluted in PBS (Table 1). Subsequently, sections were washed three times with PBS, and DNA was counterstained with 0.1 μg/mL Hoechst 33258 (Sigma-Aldrich, Milan, Italy) for 6 min, followed by PBS washes. Finally, slides were washed with PBS and mounted in Mowiol (Calbiochem-Inalco S.r.l., Milan, Italy) for fluorescence microscopy analyses. For each experimental group, three independent experiments were performed.

Fluorescence images were acquired using an Olympus BX51 microscope (EVIDENT Europe GmbH, Hamburg, Germany) equipped with 40× objectives and a 100-W mercury lamp. The following filter sets were used: 330–385 nm excitation, 400 nm dichroic mirror, and 420 nm emission for Hoechst; 450–480 nm excitation, 500 nm dichroic mirror, and 515 nm emission for Alexa Fluor 488; and 540 nm excitation, 580 nm dichroic mirror, and 620 nm emission for Alexa Fluor 594. Images were captured using an Olympus MagniFire camera system (Olympus Italia S.r.l., Segrate, Italy) and managed with Olympus Cell F software (version 3.1). Exposure times were first optimised on the control samples and subsequently kept identical across all experimental conditions to allow reliable comparison of fluorescence intensity. Quantification of fluorescence signals was then performed using ImageJ software (version 1.54j). In particular, the polygon selection tool was used to define cellular regions of interest. Labelling intensity was measured as mean intensity over the selected area, and background subtraction was performed using unstained regions from the same sections.

### 4.6. Transmission Electron Microscopy

For Transmission Electron Microscopy (TEM), neurospheres were harvested at 500 g for 6 min and then fixed with 2.5% glutaraldehyde (Cat. Nr. G5882, Sigma-Aldrich, Steinheim am Albuch, Germany) in 0.1 M phosphate buffer, pH 7.2, for 2 h at RT. After 3× washing in the same buffer, samples were post-fixed with 1% OsO_4_ (Electron Microscopy Science, Hatfield, PA, USA) in 0.1 M cacodylate buffer (Cat Nr. 12300, Electron Microscopy Sciences), pH 7.2, for 2 h at RT. Samples were dehydrated in ascending acetone (Cat N. 1300014.1000, Sigma-Aldrich) gradient (50, 75, 90, 100%) and embedded in Durcupan™ ACM resin (Cat. N. 44611-12-13, Sigma-Aldrich). After embedding, the blocks were polymerized at 60 °C for 72 h. Resin blocks were then cut using an ultramicrotome (Leica Reichert Ultracut S, Wetzlar, Germany) to obtain 70nm ultrathin sections, which were placed on pre-stained 150-mesh copper grids coated with collodion (Electron Microscopy Sciences). Before imaging, grids were contrasted with Uranyless Acetate Solution (Electron Microscopy Science) and Lead Citrate (Electron Microscopy Science), according to the manufacturer’s instructions. Images were acquired with a Talos L120C electron microscope (ThermoFisher Scientific, Waltham, MA, USA). Image resolution was 4096 × 4384, with a 1-s exposure time.

### 4.7. Statistical Analysis

Data are expressed as mean ± SEM. The normality of each dataset was assessed using the Anderson–Darling, D’Agostino–Pearson, Shapiro–Wilk, and Kolmogorov–Smirnov tests. Based on the outcome of these tests, appropriate statistical analyses were selected. When datasets met the criteria for normal distribution, differences among groups were evaluated using one-way ANOVA followed by Bonferroni’s post hoc test for multiple comparisons. For datasets that did not satisfy normality assumptions, the Kruskal–Wallis test was applied, followed by Dunn’s post hoc test. Statistical significance was defined as *p* < 0.05 (*), *p* < 0.01 (**), and *p* < 0.001 (***). For multiple comparisons, the following symbols were used: * vs. ctrl; ° vs. vehicle; $ vs. 50 µM α-PHP; # vs. 100 µM α-PHP; Ω vs. 200 µM α-PHP; Ʃ vs. EtOH. All statistical analyses were carried out using GraphPad Prism 8.0 (GraphPad Software Inc., San Diego, CA, USA).

## 5. Conclusions

Taken together, our findings demonstrate for the first time that α-PHP exerts potential neurotoxic and neuromodulatory effects in both murine NSPC differentiated under 2D cultures or 3D neurosphere model protocols. This neuronal/glial differentiation is affected by the regulation of apoptotic pathway activation, as well as the induction of morphological and ultrastructural alterations. The observed dose concentration-dependent cytotoxic effects were assessed following exposure to increasing α-PHP doses (50–200 μM) at different time points (1, 3, 7, 9, 14, 17, and 21 days in the 2D model, and after 14 days in neurospheres). Overall, α-PHP shows clear neurotoxic and neuromodulatory effects on neuronal/glial differentiation, apoptosis, and cellular ultrastructure in NSPC in vitro models.

## Figures and Tables

**Figure 2:**
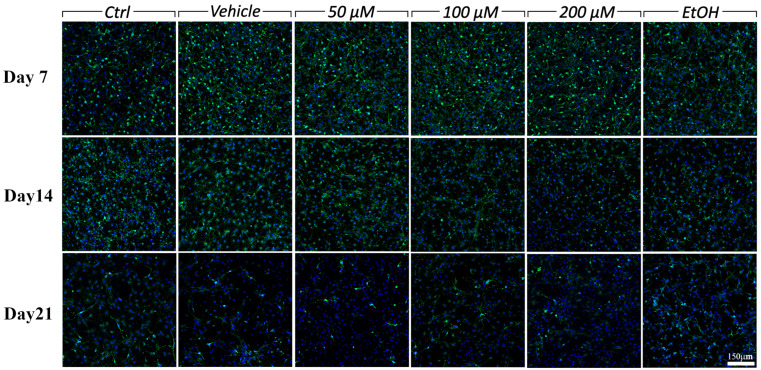
MAP2 detection in neural stem cells (NSCs) differentiated for 7, 14, or 21 days. MAP2-positive cells are shown in green, while nuclei are counterstained in blue. Scale bar 150 μm. *n* = 3–5.

**Figure 3:**
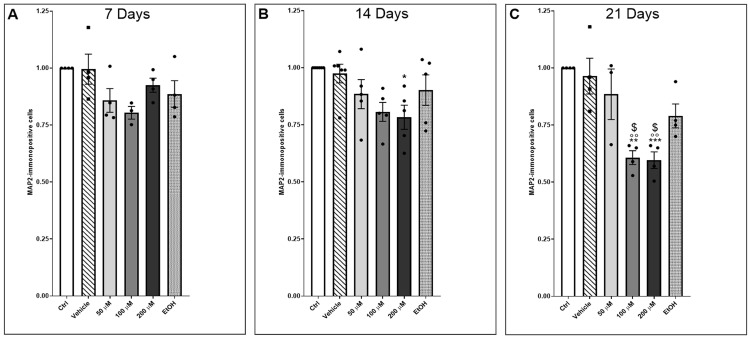
Comparison of MAP2-positive differentiated cells after exposure to different treatments for (**A**) 7 days, (**B**) 15 days, and (**C**) 21 days. For each experiment, the number of MAP2-positive cells was normalised to Ctrl. Statistically significant data: * compared to ctrl; °° compared to vehicle; $ compared to 50 µM αPHP. (*) *p* < 0.05; (**) and (°°) *p* < 0.01; (***) *p* < 0.001. *n* = 3–5.

**Table 1:** Primary and secondary antibodies are employed for immunoreactions.

	Antigen	Immunogen	Manufacturer, Species, Mono-Polyclonal, Cat., No., RRID	Dilution
Primary antibodies	MAP2	Purified antibody raised against a recombinant microtubule-associated protein	ABclonal (ABclonal Germany GmbH, Düsseldorf, Germany), Rabbit polyclonal IgG, Cat# A2572, RRID: AB_2764458	1:100
	Anti- Caspase-3 (31A1067)	Purified antibody raised against amino acids 50–86 of Caspase-3 of human origin	Santa Cruz Biotechnology (Santa Cruz, CA, USA), Mouse monoclonal IgG, Cat# sc-56053, RRID: AB_781826	1:100
	GFAP	Purified antibody raised against a 50 kDa intracytoplasmic protein of human and cow origin	Dako (Agilent Technologies Singapore, Singapore), Rabbit polyclonal IgG, Cat# Z0334, RRID: AB_10013382	1:500
Secondary antibodies	Alexa Fluor™ 488 goat anti-rabbit IgG (H + L) Highly Cross-Adsorbed Secondary Antibody	Gamma immunoglobulin heavy and light chains	Thermo Fisher Scientific (Monza, Italy)	1:200
	Alexa Fluor™ 594 goat anti-mouse IgG (H + L) Highly Cross-Adsorbed Secondary Antibody	Gamma immunoglobulin heavy and light chains	Thermo Fisher Scientific (Monza, Italy)	1:200

## Data Availability

The datasets used and analyzed during the current investigation are available in the manuscript or in the Supplementary Materials.

## Supplementary Materials

The following supporting information can be downloaded at: https://www.mdpi.com/article/10.3390/molecules31142453/s1, Table S1: Statistical analysis for the semiquantitative evaluation of MAP2-positive differentiated cells in 2D culture after exposure to different α-PHP treatments for 7 days. Data are reported as mean ± SEM. Statistical significance: (*ns*) not significant; Table S2: Statistical analysis for the semiquantitative evaluation of MAP2-positive differentiated cells in 2D culture after exposure to different α-PHP treatments for 14 days. Data are reported as mean ± SEM. Statistical significance: (*ns*) not significant, (*) *p* < 0.05; Table S3: Statistical analysis for the semiquantitative evaluation of MAP2-positive differentiated cells in 2D culture after exposure to different α-PHP treatments for 21 days. Data are reported as mean ± SEM. Statistical significance: (*ns*) not significant, (*) *p* < 0.05, (**) *p* < 0.01 and (***) *p* < 0.001; Table S4: Statistical analysis for the semiquantitative evaluation of MAP2 immunopositive optical density in 3D neutrosphere after exposure to different α-PHP treatments. Data are reported as mean ± SEM. Statistical significance: (*ns*) not significant and (***) *p* < 0.001; Table S5: Statistical analysis for the semiquantitative evaluation of Caspase 3 immunopositive optical density in 3D neutrosphere after exposure to different α-PHP treatments. Data are reported as mean ± SEM. Statistical significance: (*ns*) not significant, (*) *p* < 0.05 and (***) *p* < 0.001; Table S6: Statistical analysis for the semiquantitative evaluation of GFAP immunopositive optical density in 3D neutrosphere after exposure to different α-PHP treatments. Data are reported as mean ± SEM. Statistical significance: (*ns*) not significant, (*) *p* < 0.05, (**) *p* < 0.01 and (***) *p* < 0.001; Table S7: Statistical analysis for the semiquantitative evaluation of Caspase 3 immunopositive optical density in 3D neutrosphere after exposure to different α-PHP treatments. Data are reported as mean ± SEM. Statistical significance: (*ns*) not significant and (***) *p* < 0.001.
