# Supplementary material for: The MDPV Derivative α-PHP Regulates Cellular Differentiation and Triggers Apoptotic Cell Death and Ultrastructural Changes in Murine 3D Neurospheres
Source: Molecules. 2026 Jul 13;31(14):2453. doi: 10.3390/molecules31142453 (PMC13415019; doi:10.3390/molecules31142453)
Supplement: Supplementary file 1 [file molecules-31-02453-s001.zip › molecules-4356971-supplementary.pdf]

Supplementary material

Table S1. Statistical analysis for the semiquantitative evaluation of MAP2-positive differentiated cells in 2D culture after exposure to different α-PHP treatments for 7 days. Data are reported as mean ± SEM. Statistical significance: (*ns*) not significant.

Experimental group		Experimental group	*p*-value
Ctrl (1.00 ± 0.000)	*vs*	Vehicle (0.996± 0.066)	*ns*
Ctrl (1.00 ± 0.000)	*vs*	50 µM (0.858 ± 0.052)	*ns*
Ctrl (1.00 ± 0.000)	*vs*	100 µM (0.804 ± 0.028)	*ns*
Ctrl (1.00 ± 0.000)	*vs*	200 µM (0.925 ± 0.031)	*ns*
Ctrl (1.00 ± 0.000)	*vs*	EtOH (0.887 ± 0.058)	*ns*
Vehicle (0.996± 0.066)	*vs*	50 µM (0.858 ± 0.052)	*ns*
Vehicle (0.996± 0.066)	*vs*	100 µM (0.804 ± 0.028)	*ns*
Vehicle (0.996± 0.066)	*vs*	200 µM (0.925 ± 0.031)	*ns*
Vehicle (0.996± 0.066)	*vs*	EtOH (0.887 ± 0.058)	*ns*
50 µM (0.858 ± 0.052)	*vs*	100 µM (0.804 ± 0.028)	*ns*
50 µM (0.858 ± 0.052)	*vs*	200 µM (0.925 ± 0.031)	*ns*
50 µM (0.858 ± 0.052)	*vs*	EtOH (0.887 ± 0.058)	*ns*
100 µM (0.804 ± 0.028)	*vs*	200 µM (0.925 ± 0.031)	*ns*
100 µM (0.804 ± 0.028)	*vs*	EtOH (0.887 ± 0.058)	*ns*
200 µM (0.925 ± 0.031)	*vs*	EtOH (0.887 ± 0.058)	*ns*

Table S2. Statistical analysis for the semiquantitative evaluation of MAP2-positive differentiated cells in 2D culture after exposure to different α-PHP treatments for 14 days. Data are reported as mean ± SEM. Statistical significance: (*ns*) not significant, (*) $p < 0.05$.

Experimental group		Experimental group	*p*-value
Ctrl (1.00 ± 0.000)	*vs*	Vehicle (0.975 ± 0.041)	*ns*
Ctrl (1.00 ± 0.000)	*vs*	50 µM (0.885 ± 0.064)	*ns*
Ctrl (1.00 ± 0.000)	*vs*	100 µM (0.807 ± 0.041)	*ns*
Ctrl (1.00 ± 0.000)	*vs*	200 µM (0.783 ± 0.053)	*
Ctrl (1.00 ± 0.000)	*vs*	EtOH (0.902 ± 0.067)	*ns*
Vehicle (0.975 ± 0.041)	*vs*	50 µM (0.885 ± 0.064)	*ns*
Vehicle (0.975 ± 0.041)	*vs*	100 µM (0.807 ± 0.041)	*ns*

Vehicle (0.975 ± 0.041)	*vs*	200 µM (0.783 ± 0.053)	*ns*
Vehicle (0.975 ± 0.041)	*vs*	EtOH (0.902 ± 0.067)	*ns*
50 µM (0.885 ± 0.064)	*vs*	100 µM (0.807 ± 0.041)	*ns*
50 µM (0.885 ± 0.064)	*vs*	200 µM (0.783 ± 0.053)	*ns*
50 µM (0.885 ± 0.064)	*vs*	EtOH (0.902 ± 0.067)	*ns*
100 µM (0.807 ± 0.041)	*vs*	200 µM (0.783 ± 0.053)	*ns*
100 µM (0.807 ± 0.041)	*vs*	EtOH (0.902 ± 0.067)	*ns*
200 µM (0.783 ± 0.053)	*vs*	EtOH (0.902 ± 0.067)	*ns*

Table S3. Statistical analysis for the semiquantitative evaluation of MAP2-positive differentiated cells in 2D culture after exposure to different α-PHP treatments for 21 days. Data are reported as mean ± SEM. Statistical significance: (*ns*) not significant, (*) $p < 0.05$, (**) $p < 0.01$ and (***) $p < 0.001$.

**Experimental group**		**Experimental group**	***p*-value**
Ctrl (1.000 ± 0.000)	*vs*	Vehicle (0.965 ± 0.078)	*ns*
Ctrl (1.000 ± 0.000)	*vs*	50 µM (0.885 ± 0.111)	*ns*
Ctrl (1.000 ± 0.000)	*vs*	100 µM (0.607 ± 0.030)	**
Ctrl (1.000 ± 0.000)	*vs*	200 µM (0.596 ± 0.037)	***
Ctrl (1.000 ± 0.000)	*vs*	EtOH (0.790 ± 0.052)	*ns*
Vehicle (0.965 ± 0.078)	*vs*	50 µM (0.885 ± 0.111)	*ns*
Vehicle (0.965 ± 0.078)	*vs*	100 µM (0.607 ± 0.030)	**
Vehicle (0.965 ± 0.078)	*vs*	200 µM (0.596 ± 0.037)	**
Vehicle (0.965 ± 0.078)	*vs*	EtOH (0.790 ± 0.052)	*ns*
50 µM (0.885 ± 0.111)	*vs*	100 µM (0.607 ± 0.030)	*
50 µM (0.885 ± 0.111)	*vs*	200 µM (0.596 ± 0.037)	*
50 µM (0.885 ± 0.111)	*vs*	EtOH (0.790 ± 0.052)	*ns*
100 µM (0.607 ± 0.030)	*vs*	200 µM (0.596 ± 0.037)	*ns*
100 µM (0.607 ± 0.030)	*vs*	EtOH (0.790 ± 0.052)	*ns*
200 µM (0.596 ± 0.037)	*vs*	EtOH (0.790 ± 0.052)	*ns*

Table S4. Statistical analysis for the semiquantitative evaluation of MAP2 immunopositive optical density in 3D neutrosphere after exposure to different α-PHP treatments. Data are reported as mean ± SEM. Statistical significance: (*ns*) not significant and (***) $p < 0.001$.

Experimental group		Experimental group	*p*-value
Ctrl (66.52 ± 4.36)	*vs*	Vehicle (67.35 ± 2.59)	*ns*
Ctrl (66.52 ± 4.36)	*vs*	50 µM (77.46 ± 2.36)	*ns*
Ctrl (66.52 ± 4.36)	*vs*	100 µM (77.12 ± 2.80)	*ns*
Ctrl (66.52 ± 4.36)	*vs*	200 µM (88.00 ± 1.77)	***
Ctrl (66.52 ± 4.36)	*vs*	EtOH (88.33 ± 4.50)	***
Vehicle (67.35 ± 2.59)	*vs*	50 µM (77.46 ± 2.36)	*ns*
Vehicle (67.35 ± 2.59)	*vs*	100 µM (77.12 ± 2.80)	*ns*
Vehicle (67.35 ± 2.59)	*vs*	200 µM (88.00 ± 1.77)	***
Vehicle (67.35 ± 2.59)	*vs*	EtOH (88.33 ± 4.50)	***
50 µM (77.46 ± 2.36)	*vs*	100 µM (77.12 ± 2.80)	*ns*
50 µM (77.46 ± 2.36)	*vs*	200 µM (88.00 ± 1.77)	*ns*
50 µM (77.46 ± 2.36)	*vs*	EtOH (88.33 ± 4.50)	*ns*
100 µM (77.12 ± 2.80)	*vs*	200 µM (88.00 ± 1.77)	*ns*
100 µM (77.12 ± 2.80)	*vs*	EtOH (88.33 ± 4.50)	*ns*
200 µM (88.00 ± 1.77)	*vs*	EtOH (88.33 ± 4.50)	*ns*

Table S5. Statistical analysis for the semiquantitative evaluation of Caspase 3 immunopositive optical density in 3D neutrosphere after exposure to different α-PHP treatments. Data are reported as mean ± SEM. Statistical significance: (*ns*) not significant, (*) $p < 0.05$ and (***) $p < 0.001$.

Experimental group		Experimental group	*p*-value
Ctrl (5.59 ± 1.20)	*vs*	Vehicle (6.48 ± 1.12)	*ns*
Ctrl (5.59 ± 1.20)	*vs*	50 µM (118.71 ± 2.49)	***
Ctrl (5.59 ± 1.20)	*vs*	100 µM (133.64 ± 2.84)	***
Ctrl (5.59 ± 1.20)	*vs*	200 µM (134.17 ± 7.86)	***
Ctrl (5.59 ± 1.20)	*vs*	EtOH (136.39 ± 3.20)	***
Vehicle (6.48 ± 1.12)	*vs*	50 µM (118.71 ± 2.49)	***
Vehicle (6.48 ± 1.12)	*vs*	100 µM (133.64 ± 2.84)	***
Vehicle (6.48 ± 1.12)	*vs*	200 µM (134.17 ± 7.86)	***

Vehicle (6.48 ± 1.12)	*vs*	EtOH (136.39 ± 3.20)	***
50 µM (118.71 ± 2.49)	*vs*	100 µM (133.64 ± 2.84)	*ns*
50 µM (118.71 ± 2.49)	*vs*	200 µM (134.17 ± 7.86)	*ns*
50 µM (118.71 ± 2.49)	*vs*	EtOH (136.39 ± 3.20)	*
100 µM (133.64 ± 2.84)	*vs*	200 µM (134.17 ± 7.86)	*ns*
100 µM (133.64 ± 2.84)	*vs*	EtOH (136.39 ± 3.20)	*ns*
200 µM (134.17 ± 7.86)	*vs*	EtOH (136.39 ± 3.20)	*ns*

Table S6. Statistical analysis for the semiquantitative evaluation of GFAP immunopositive optical density in 3D neutrosphere after exposure to different α-PHP treatments. Data are reported as mean ± SEM. Statistical significance: (*ns*) not significant, (*) $p < 0.05$, (**) $p < 0.01$ and (***) $p < 0.001$.

**Experimental group**		**Experimental group**	***p*-value**
Ctrl (105.88 ± 3.88)	*vs*	Vehicle (106.53 ± 3.21)	*ns*
Ctrl (105.88 ± 3.88)	*vs*	50 µM (144.65 ± 5.72)	***
Ctrl (105.88 ± 3.88)	*vs*	100 µM (123.07 ± 2.38)	*
Ctrl (105.88 ± 3.88)	*vs*	200 µM (93.07 ± 1.46)	*
Ctrl (105.88 ± 3.88)	*vs*	EtOH (60.09 ± 1.03)	***
Vehicle (106.53 ± 3.21)	*vs*	50 µM (144.65 ± 5.72)	***
Vehicle (106.53 ± 3.21)	*vs*	100 µM (123.07 ± 2.38)	**
Vehicle (106.53 ± 3.21)	*vs*	200 µM (93.07 ± 1.46)	*ns*
Vehicle (106.53 ± 3.21)	*vs*	EtOH (60.09 ± 1.03)	***
50 µM (144.65 ± 5.72)	*vs*	100 µM (123.07 ± 2.38)	***
50 µM (144.65 ± 5.72)	*vs*	200 µM (93.07 ± 1.46)	***
50 µM (144.65 ± 5.72)	*vs*	EtOH (60.09 ± 1.03)	***
100 µM (123.07 ± 2.38)	*vs*	200 µM (93.07 ± 1.46)	***
100 µM (123.07 ± 2.38)	*vs*	EtOH (60.09 ± 1.03)	***
200 µM (93.07 ± 1.46)	*vs*	EtOH (60.09 ± 1.03)	***

Table S7. Statistical analysis for the semiquantitative evaluation of Caspase 3 immunopositive optical density in 3D neutrosphere after exposure to different α-PHP treatments. Data are reported as mean ± SEM. Statistical significance: (*ns*) not significant and (***) $p < 0.001$.

**Experimental group**		**Experimental group**	***p*-value**
Ctrl (8.35 ± 1.49)	*vs*	Vehicle (9.61 ± 2.08)	*ns*
Ctrl (8.35 ± 1.49)	*vs*	50 µM (147.94 ± 3.07)	***
Ctrl (8.35 ± 1.49)	*vs*	100 µM (152.26 ± 3.87)	***
Ctrl (8.35 ± 1.49)	*vs*	200 µM (151.72 ± 7.09)	***
Ctrl (8.35 ± 1.49)	*vs*	EtOH (157.21 ± 2.34)	***
Vehicle (9.61 ± 2.08)	*vs*	50 µM (147.94 ± 3.07)	***
Vehicle (9.61 ± 2.08)	*vs*	100 µM (152.26 ± 3.87)	***
Vehicle (9.61 ± 2.08)	*vs*	200 µM (151.72 ± 7.09)	***
Vehicle (9.61 ± 2.08)	*vs*	EtOH (157.21 ± 2.34)	***
50 µM (147.94 ± 3.07)	*vs*	100 µM (152.26 ± 3.87)	*ns*
50 µM (147.94 ± 3.07)	*vs*	200 µM (151.72 ± 7.09)	*ns*
50 µM (147.94 ± 3.07)	*vs*	EtOH (157.21 ± 2.34)	*ns*
100 µM (152.26 ± 3.87)	*vs*	200 µM (151.72 ± 7.09)	*ns*
100 µM (152.26 ± 3.87)	*vs*	EtOH (157.21 ± 2.34)	*ns*
200 µM (151.72 ± 7.09)	*vs*	EtOH (157.21 ± 2.34)	*ns*
